# Kawasaki Disease Complicated by Late-Onset Fatal Cerebral Infarction: A Case Report and Literature Review

**DOI:** 10.3389/fped.2021.598867

**Published:** 2021-05-19

**Authors:** Lin Wang, Hongyu Duan, Kaiyu Zhou, Yimin Hua, Xiaoliang Liu, Chuan Wang

**Affiliations:** ^1^Key Laboratory of Birth Defects and Related Diseases of Women and Children, Ministry of Education Chengdu, Sichuan University, Chengdu, China; ^2^Department of Pediatric Cardiology, West China Second University Hospital, Sichuan University, Chengdu, China; ^3^The Cardiac Development and Early Intervention Unit, West China Institute of Women and Children's Health, West China Second University Hospital, Sichuan University, Chengdu, China; ^4^Key Laboratory of Development and Diseases of Women and Children of Sichuan Province, West China Second University Hospital, Sichuan University, Chengdu, China

**Keywords:** kawasaki disease, coronary artery aneurysms, thrombosis, late-onset, cerebral infarction

## Abstract

**Background:** Cerebral infarction is a rare neurological complication of Kawasaki disease (KD) and occurs in the acute or subacute stage. There have been no reported cases of late-onset fatal cerebral infarction presenting over 1 year after the onset of KD.

**Case Presentation:** A 5-month-old male patient with KD received timely intravenous immunoglobulin therapy; however, extensive coronary artery aneurysms (CAA) and coronary artery thrombosis (CAT) developed 1 month later. Anticoagulation and thrombolytic agents were suggested, but the child's parents refused. Fifteen months after KD onset, an attack of syncope left him with left hemiplegia; brain computerized tomography (CT) scans revealed cerebral infarction of the right basal ganglion without hemorrhage. Magnetic resonance angiography (MRA) revealed severe stenosis of the right middle cerebral artery, and a series of tests were performed to exclude other causes of cerebral infarction. Considering the cerebral infarction and CAT, combination therapy with urokinase and low-molecular-weight heparin (LMWH) was initiated within 24 h of syncope onset, together with oral aspirin and clopidogrel. Five days later, his clinical symptoms partially regressed and he was discharged. Unfortunately, 5 days after discharge, his clinical condition suddenly deteriorated. Repeat brain CT showed hemorrhagic stroke involving the entire left cerebral area, in addition to the previous cerebral infarction in the right basal ganglion, with obvious secondary cerebral swelling and edema, which might have been caused by previous thrombolysis. Severe cerebral hernias developed quickly. Regrettably, the patient's parents abandoned treatment because of economic factors and unfavorable prognosis, and he died soon after.

**Conclusions:** Cerebral infarction and cerebral artery stenosis can develop late, even 1 year after the onset of KD. Pediatricians should be aware of the possibility of cerebrovascular involvement in addition to cardiac complications during long-term follow-up of KD patients. Prompt anticoagulation therapy and regular neuroimaging evaluation are essential for the management of patients with KD with giant CAA and/or CAT.

## Background

Kawasaki disease (KD) is an acute vasculitis of unknown etiology that mainly affects children younger than 5 years of age (1). Although intravenous immunoglobulin (IVIG) treatment is quite effective, ~4% of patients still develop coronary artery aneurysms (CAA), stenosis, or occlusion (2). Moreover, other medium-sized arteries may also be involved in patients with KD, affecting multiple organs and tissues such as those of the pulmonary and gastrointestinal systems (3–5). Acute involvement of the central nervous system has also been described and is being increasingly reported in the literature (6–11). The most frequently described neurological manifestations are headache, convulsions, somnolence, extreme irritability, signs of meningeal irritation, bulging fontanelles, hemiparesis, and facial palsy (12, 13). Cerebral infarction has been described as a rare complication of KD and always occurs during acute or subacute illness (14–25). Currently, only one case of late-onset cerebral infarction has been reported, occurring 4 months after KD onset (16).

Herein, we report the case of a 5-month-old infant with KD, who received timely IVIG treatment. Giant CAA and coronary artery thrombosis (CAT) still developed 1 month after disease onset, and cerebral infarction occurred 15 months later. To the best of our knowledge, our patient is the first reported case of very late-onset fatal cerebral infarction, appearing over 1 year after an acute episode of KD. This suggests that apart from cardiac complications, pediatricians should be alert to the possibility of cerebral infarction during long-term follow-up of patients with KD.

## Case Presentation

**Figure 1:**
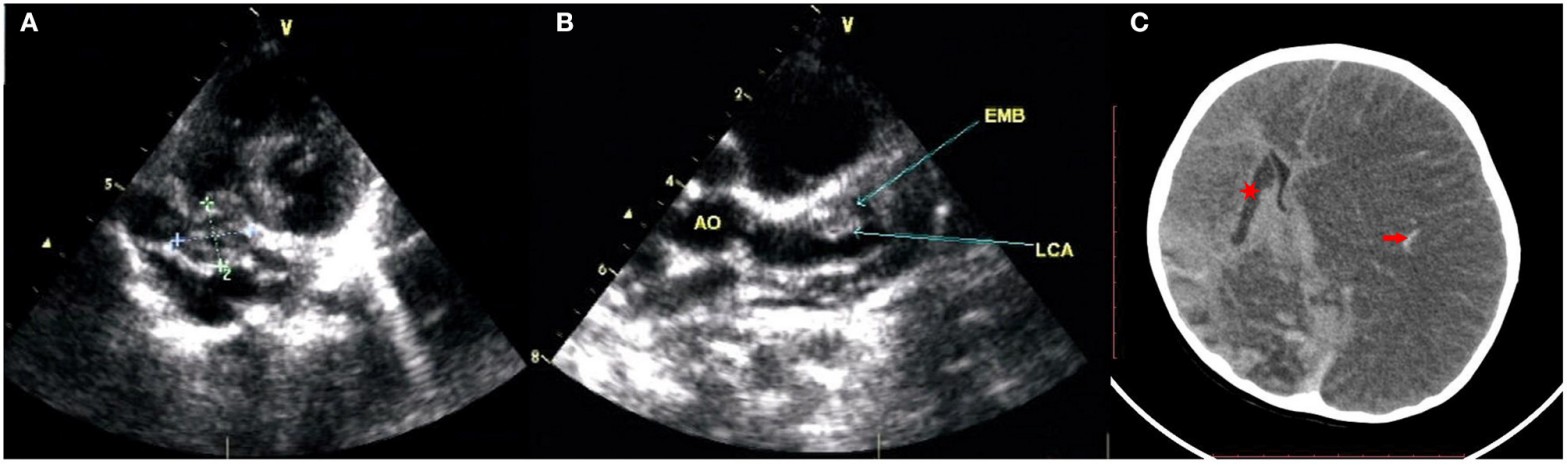
The echocardiography revealed extensive CAA and CAT. **(A)** Right coronary artery aneurysms sized 9 mm and thrombosis sized 19 mm × 14 mm; **(B)** left anterior descending artery aneurysms sized 11 mm and thrombosis sized 11 × 9 mm, left circumflex coronary artery aneurysms sized 14 mm. **(C)** The brain CT showed cerebral infarction of right basal ganglion (red star), hemorrhagic stroke involved in an entire left cerebral area with obviously secondary cerebral swelling, edema (red arrow).

A previously healthy male infant aged 5 months was diagnosed with KD, characterized by the presence of fever ≥5 days, rash, conjunctivitis, oral changes, and cervical lymphadenopathy, at the local hospital. On the 6 day after the onset of fever, he received IVIG (2 g/kg, single intravenous infusion) and aspirin (30 mg/kg/day) treatment. Encouragingly, he defervesced within 48 h of IVIG infusion, and the dose of aspirin was tapered (5 mg/kg/day). During this local hospitalization, coronary artery lesions (CAL) were not detected on echocardiography performed once a week. One month later, the fourth repeat echocardiography revealed extensive CAA, involving 9 mm of the right coronary artery (RCA), 11 mm of the left anterior descending artery (LAD), and 14 mm of the left circumflex coronary artery (LCA), even though the child had been prescribed maintenance aspirin therapy (5 mg/kg/day). CAT was also identified, measuring 19 mm × 14 mm in the RCA and 11 × 9 mm in the LAD. Thereafter, he was transferred to our hospital and visited a pediatric cardiologist at the outpatient department with unremarkable clinical symptoms. Owing to the presence of CAT and the rapid progression of CAL, anticoagulation (warfarin) and thrombolytic agents (low-molecular-weight heparin (LMWH) or tissue-type plasminogen activator) were suggested, but the child's parents refused due to the inconvenience of monitoring international normalized ratios (INR) and underlying bleeding complications, despite the necessity of thrombolysis therapy being repeatedly explained to them. Only clopidogrel (1 mg/kg/day) with aspirin (5 mg/kg/day) was agreed on and maintained during subsequent follow-up at our outpatient department, and both CAT and CAA remained unchanged without progression or regression (Figures 1A,B).

Fifteen months after KD onset, he suffered a sudden syncope attack without obvious triggers, and he was immediately transferred to a local hospital. On arrival, he was lethargic but afebrile and had no bradycardia, irregular breath, or hypoxia. His blood pressure was also normal. Physical examination was only remarkable for left hemiplegia with a muscle motor power grade I/V in the left extremities and grade V/V in the right extremities. History of head and neck trauma was ruled out by the child's parents.

An emergent brain computerized tomography (CT) scan was immediately performed and revealed cerebral infarction of the right basal ganglion without hemorrhage. Meanwhile, owing to the history of KD, echocardiography was also conducted, which only revealed the unchanged CAA and CAT without other remarkable findings. Thereafter, a series of targeted tests were conducted to explore the underlying etiologies of cerebral infarction. First, intracranial or systemic infections were not considered because no fever was reported during the disease course and all values of complete blood count, C-reactive protein (CRP), and procalcitonin (PCT) were normal, and the results of microorganism examinations such as sputum culture, TORCH, mycoplasma immunoglobulin M (MP-IgM), SPOT-Tuberculosis test (T-SPOT), and purified protein derivative (PPD) skin test were negative. Second, other common causes of cerebral infarction including sickle cell disease, thrombophilic states, connective tissue diseases (e.g., systemic lupus erythematosus), and vasculitis (e.g., nodular polyarteritis, antineutrophil cytoplasmic antibodies (ANCA)-associated vasculitis) were also excluded due to unremarkable results from complete blood count, reticulocyte count, CRP level, erythrocyte sedimentation rate, hemoglobin electrophoresis, liver function, renal function, blood lipid level, autoantibody, ANCA, anticardiolipin antibody, serum complement level, and disseminated intravascular coagulation test. Lastly, severe stenosis of the right middle cerebral artery was found on magnetic resonance angiography (MRA). Based on these findings, it was proposed that cerebral infarction in our patient was most likely related to the ongoing cerebral vasculitis associated with KD since similar cases had been reported before (18, 20, 21). However, it was a great pity that we could only obtain the brain CT and MRA reports and not the images due to copyright issues.

Considering the cerebral infarction and CAT, combination therapy with intravenous urokinase (4 × 10^3^ U/kg/day for 3 days) and subcutaneous LMWH (85 IU/kg every 12 h) was initiated within 24 h of syncope onset, together with oral aspirin (3 mg/kg/day) and clopidogrel (1 mg/kg/day). The child was also required to limit activities and stay in bed, while undertaking other general measures including maintaining the head in the midline position, maintaining normal body temperature and glycemia, mannitol, and hyperosmolar therapy. Five days later, his clinical symptoms had partially regressed and he was discharged home, although imaging findings showed that his CAT and cerebral infarction had not regressed. Rehabilitation training was prescribed on an outpatient basis.

**Figure 2:**
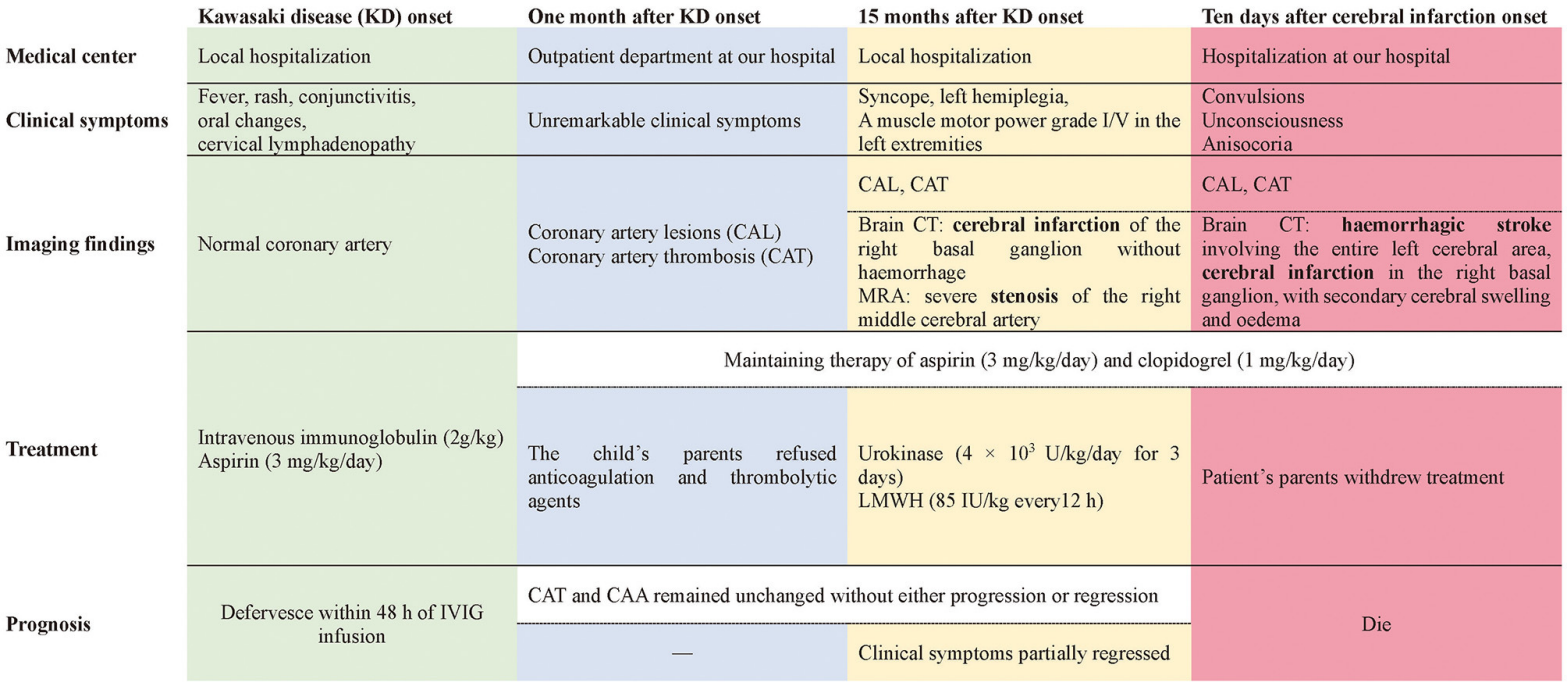
Clinical course of this patient.

Unfortunately, 5 days after discharge, his clinical condition suddenly deteriorated, with convulsions, unconsciousness, and anisocoria. The patient was admitted to our hospital. Repeat brain CT showed hemorrhagic stroke involving the entire left cerebral area in addition to the previous cerebral infarction in the right basal ganglion, with obvious secondary cerebral swelling and edema (Figure 1C), which might have resulted from bleeding complications from previous thrombolysis therapy. Severe cerebral hernia developed quickly. Regrettably, the patient's parents withdrew treatment because of economic factors and the unfavorable prognosis. Unfortunately, the child died soon after (Figure 2).

## Discussion

Cerebral infarction is a rare neurological complication in patients with KD, and it always occurs during the acute or subacute stage. In the literature, there has been only one case of late-onset ischemic stroke described 4 months after onset of KD (14–26). To our knowledge, our patient presented the longest interval between KD onset and fatal cerebral infarction (15 months). This case strongly suggests that pediatricians should be alert to the possibility of late-onset cerebral infarction in patients with KD, and that anticoagulation therapy is very important and essential in patients with CAA and/or CAT, especially in those with severe complications and/or progression. Neuroimaging might be recommended for long-term management of these patients, as it is necessary.

Although there is no direct evidence that the cerebral infarction in our case was caused by KD, there is substantial indirect evidence supporting a causal relationship. First, after the diagnosis of cerebral infarction was established, a series of targeted tests were conducted to uncover the underlying etiologies. The common causes of cerebral infarction, including infectious diseases, head and neck trauma, cardiogenic embolism, sickle cell disease, prothrombotic states, connective tissue diseases (e.g., systemic lupus erythematosus), and other vasculitis (e.g., nodular polyarteritis, ANCA-associated vasculitis) were excluded. The possibility of moyamoya disease and syndrome was also excluded by MRA. Second, despite its rarity, KD-associated cerebral infarction has been reported in several cases (14–26). In most cases, cerebral infarction always occurred in the middle cerebral artery territory because KD predominantly affects medium-sized arteries (14, 15, 17, 21, 23–27). In our patient, the initial brain CT showed a well-defined hypodense lesion involving gray and white matter in the right basal ganglion, suggestive of right middle cerebral artery infarction. Accordingly, severe stenosis of the right cerebral artery was observed on MRA. Third, aneurysms and/or stenosis have been reported in other extracardiac muscular arteries, including cerebral arteries, in patients with KD, and their prognosis seems to resemble that of CAA (28–30). A recent study identified that giant CAA and progression of CAL were risk factors for developing systemic artery aneurysms (SAAs) in KD. All patients with SAAs had concomitant CAA with *z*-scores >8, of which 73.9% had giant CAA (30). Given the giant CAA and rapid progression of CAL in our patient, it is conceivable that the cerebral arteries were most likely involved. Furthermore, anticoagulation therapy (warfarin) had been initially refused by the child's parents due to the inconvenience of monitoring of INR and underlying bleeding complications, despite the necessity of thrombolysis therapy being repeatedly explained to them, which may have increased the risk of thrombosis in the affected cerebral arteries and thereby led to cerebral infarction in our case.

In terms of the underlying mechanisms with respect to cerebral infarction in KD, several potential explanations have been proposed, although it is not entirely clear. First, thrombotic complications are a well-known complication of immunoglobulin therapy and may be related to increased blood viscosity, the procoagulant activity of immunoglobulin itself, and immunoglobulin-induced arterial vasospasm (31). IVIG-associated thrombosis, which frequently develops within 24 h after IVIG infusion, has also been suggested as a cause of cerebral infarction in KD and has been previously reported in two cases (17, 19). However, cerebral infarction occurred in our patient more than 1 year after IVIG treatment; therefore, it was not considered in our case. However, it is notable that pediatricians should be alert to clinical symptoms of cerebral infarction within 24 h of IVIG infusions. Second, embolisms induced by myocardial infarction likely provoke cerebral infarction in the course of KD (32), particularly in those with patent foramen ovale (PFO). Nevertheless, CAT in our patient persisted for more than 1 year, and it was most likely organized (33, 34). Furthermore, no clinical symptoms or signs related to myocardial infarction have been observed during the disease course. Most importantly, no PFO or intracavity embolism was noted on echocardiography. Therefore, cardiogenic embolism may not have been the cause in our patient. Additionally, cerebral vasculitis has also been proposed as one of the core underlying mechanisms associated with ischemic stroke in KD. Autopsies of children with KD have revealed signs of cerebral vasculitis affecting small- and medium-sized vessels, with features of endarteritis, periarteritis, and perivascular cuffing (35). Occlusion/stenosis and/or aneurysm of the cerebral arteries were observed in patients with KD complicated by cerebral infarction (15–17, 21, 25). Additionally, a very specific radiological sign that strengthened the vasculitis hypothesis was diffuse microhemorrhages (T2^*^-weighted sequence) associated with white matter injury on brain magnetic resonance imaging (MRI) in a 4-year-old girl who presented with unusual, rapidly catastrophic KD in which tetraplegia developed 15 days after KD onset (18). Moreover, single-photon emission computed tomography (SPECT) study showed asymptomatic focal hypoperfusion areas in 29% of patients with KD, suggesting a vasculitis mechanism (36). Based on the aforementioned evidence and the severe stenosis of the right middle cerebral artery in our case, the ongoing intracranial artery vasculitis associated with KD more likely contributed to the development of cerebral infarction in our patient.

According to the scientific statement regarding management of stroke in neonates and children from the American Heart Association/American Stroke Association (38), the primary prevention of cerebral infraction in children was difficult because the underlying causes are diverse and far different from the commonly occurring risk factors for adult stroke. However, as aforementioned, KD patients with large/giant CAA and/or CAT seemed to possess a higher risk for the occurrence of cerebral infraction since these patients may suffer from intracardiac thrombosis induced by myocardial infarction and/or ongoing intracranial artery vasculitis. Therefore, KD patients complicated with large/giant CAA should be given prompt and adequate systematic anticoagulation with LMWH or warfarin (international normalized ratio target: 2.0–3.0) in addition to low-dose aspirin (1, 39), which may reduce the risk of coronary artery thrombosis and could possibly in turn prevent the occurrence of cerebral infarction. In addition, regular neuroimaging evaluation such as MRA for these high-risk patients might also be performed to detect abnormal changes in the nervous system timely.

To date, only 13 cases of cerebral infarction in KD have been reported, manifesting as seizures, hemiparesis, motor aphagia, or disturbance of consciousness (Table 1). These patients were aged 4–48 months (mean age: 25.2 months), and only one patient was younger than 6 months old. The male-to-female ratio was 3:1. Of these patients, 75.0% (9/12) presented with typical clinical features of KD, 75.0% (9/12) had received IVIG administration, five of these nine patients still had developed CALs, and three developed other artery aneurysms [including carotid occlusion (17), large aneurysms in the axillary and internal iliac arteries bilaterally (22), and widespread clusters of fusiform or saccular aneurysms of abdominal vessel (25)]. Based on these case series, it seemed difficult to identify the clinical predictors of cerebral infarction in KD. However, it seemed that all patients had a prolonged fever during the acute phase of KD, as the mean fever duration was 18.2 days (range, 7–57 days). Moreover, a devastating course developed in most cases. These findings suggest that clinicians should monitor for cerebrovascular involvement as well as cardiovascular complications in KD patients with prolonged fever or severe progression. Most importantly, asymptomatic cerebral infarction was also found in two patients with KD, which was identified by brain MRI and/or SPECT (20, 22). Thus, the incidence of neurological involvement may be much higher than that recognized by clinicians. With respect to potentially fatal prognosis and difficulties in the management of neurological involvement, clinicians should be aware of late-onset neurological complications, particularly for cerebral infarction in patients with KD, which has been widely recognized. Artery angiography should be considered necessary, especially in patients with giant CAA and/CAT (30).

**Table 1:** Literature review of cerebral infarction in 13 patients with kawasaki disease.

**References**	**Sex/Age (month)**	**Diagnostic criteria of KD^#^**	**Fever days**	**Symptoms of Cerebral infarction**	**Cerebral artery lesions**	**Coronary artery lesions**	**Severe presentations**	**Initial therapy of KD**	**Anticoagulation/ thrombolytic therapy**	**Prognosis**
Lapointe et al. (25)	M/4	1,4,5,6	21	Seizure, hemiparesis at 6th week	Occlusion of the parietal and temporo-occipital branches of right MCA	Giant CAA	SAAs	Prednisone at 3rd week; Azathioprine at 6th week	–	Regression and CAD at 8^th^ week
Laxer et al. (24)	F/26	1,2,3,5,6	10	Seizure and hemiparesis at 5th day	Slow filling of anterior branch of left MCA	–	–	Prednisone at 6^th^ day	–	Regression at 8^th^ week
Philip (23)	-/6	1,5	21	Seizure at 4th week	A massive infarction in the distribution of right MCA	CAA	Dilated and poorly contractive LV	Not receive IVIG	–	Died of myocardial ischemia
Fujiwara et al. (22)	M/22	1,2,3,4,5,6	57	Asymptomatic	Involve right caudate nucleus and putamen	Giant CAA	DIC, SAAs	IVIG at 6th day	Not applied	Alive
Suda et al. (21)	M/8	–	20	Hemiparesis at 20th day	Obstruction of left MCA	Giant CAA, CAT	–	IVIG, aspirin at 20th day	Heparin, warfarin, PCI twice	Thrombosis in LCX
Muneuchi et al. (20)	M/48	1,2,3,4,5,6	11	Asymptomatic	Obliteration of right PICA	CAD	–	IVIG/aspirin at 5th day; methylpredonisolone at 11th day	Heparin at 11th day	No regression
Wada et al. (19)	M/36	1,2,3,4,5	15	Motor aphagia and hemiplegia at 10^th^ day	Left cerebral infarction in the parieto-temporal lobe and the left basal ganglia	Normal	–	IVIG, aspirin at 6th day	Not applied	Clinical remission
Gitiaux et al. (18)	M/48	1,2,3,4,6	13	Tetraplegia and disturbance of consciousness at 15^th^ day	Diffuse ischemic damage with microhemorrhage (vasculitis)	CALs	Severe multi-organ involvement	IVIG, aspirin at 6th day; methylprednisolone at 8th day followed by cyclophosphamide	Not applied	Clinical remission
Wang et al. (26)	M/18	1,2,3,4,5	26	Seizure at 20th day	Infarction in the distribution of MCA	Normal	–	IVIG, aspirin at 24th day	Not applied	Seizure
Sabatier et al. (17)	F/18	1,2,3,4,5,6	10	Hemiplegia and a left ptosis at 11th day	Occlusion of left MCA	Normal	Carotid occlusion	IVIG, aspirin at 10th day	Enoxaparin	Right hemiplegia
Tassinari et al. (16)	F/31	1,2,3,4,5,6	7	Hemiplegia and facial palsy at 4th month after KD	Cerebral infarction in the absence of thrombosis or aneurysms of medium and large-vessels	Normal	–	IVIG, aspirin at 7th day	Not applied	Clinical remission
Prangwatanagul et al. (15)	M/15	1,2,3,4,5	18	Hemiplegia at 15th day	Segmental mild stenosis of branch of the right MCA	CAA	–	Not receive IVIG	–	-
Nikkhah (14)	M/48	1,3,6	8	hemiparesis and aphasia at 3rd day	Complete obliteration of the left MCA	CAA?	–	IVIG and aspirin	Not applied	Clinical remission
Present case (37)	M/20	1,2,3,5	7	Seizure and hemiparesis at 15th month after KD	Stenosis of the right middle cerebral artery	Giant CAA, CAT	–	IVIG, aspirin and clopidogrel	Low molecular weight heparin, urokinase	Die

# *Diagnostic criteria of KD includes 1. Fever for at least 5 days; 2. Bilateral bulbar conjunctival injection without exudate; 3. Changes in lips and oral cavity; 4. Changes in extremities; 5. Polymorphous exanthem; 6. Cervical lymphadenopathy*.

*CAA, coronary artery aneurysms; CAT, coronary artery thrombosis; MCA: middle cerebral artery; M, male; F, female; KD, Kawasaki disease; IVIG, intravenous immunoglobulin; SAA, systemic artery aneurysms; DIC, disseminated intravascular coagulation; PICA, posterior inferior cerebellar artery; LV, left ventricle*.

## Conclusions

Cerebral infarction and cerebral artery stenosis are a substantial rarity in the complications of KD, and it could present late, even over 1 year after KD onset. Given its fatal prognosis, pediatricians should be aware of the possibility of cerebral vascular involvement in addition to cardiac complications during the long-term follow-up of patients with KD. Our case highlights the importance of prompt anticoagulation therapy and suggests that regular neuroimaging evaluation might be essential for the management of patients with KD, particularly those with giant CAA and/or CAT.

## Data Availability Statement

The original contributions presented in the study are included in the article/Supplementary Material, further inquiries can be directed to the corresponding authors.

## Ethics Statement

Written informed consent was obtained from the minors' legal guardian/next of kin for the publication of any potentially identifiable images or data included in this article.

## Author Contributions

KYZ, YMH, XLL, and CW participated in the research design and revised the manuscript. HYD and LW drafted the manuscript. LW provided the table and figures. All authors confirmed revisions and approved the final manuscript as submitted, approved the final manuscript as submitted, and agreed to be accountable for all aspects of the work.

## Conflict of Interest

The authors declare that the research was conducted in the absence of any commercial or financial relationships that could be construed as a potential conflict of interest.

## Abbreviations

ANCA: antineutrophil cytoplasmic antibodies
CALs: coronary artery lesions
CAA: coronary artery aneurysms
CAT: coronary artery thrombosis
CAL: coronary artery lesions
CT: computerized tomography
CRP: C reaction protein
INR: international normalized ratio
IVIG: intravenous immunoglobulin
INR: international ratio
KD: Kawasaki disease
LAD: left anterior descending artery
LCA: left circumflex coronary artery
LMWH: low molecular weight heparin
MRA: magnetic resonance angiography
MRI: magnetic resonance imaging
MP-IgM: mycoplasma immunoglobulin M
PCT: procalcitonin
PPD: purified protein derivative
RCA: right coronary artery
SAAs: systemic artery aneurysms
T-SPOT: SPOT-Tuberculosis test
SPECT: single-photon emission computed tomography
PFO: patent foramen ovale.
