# Supplementary material for: Kawasaki Disease Complicated by Late-Onset Fatal Cerebral Infarction: A Case Report and Literature Review
Source: Front Pediatr. 2021 May 19;9:598867. doi: 10.3389/fped.2021.598867 (PMC8170391; doi:10.3389/fped.2021.598867)
Supplement: Supplementary file 1 [file Data_Sheet_1.PDF]

CARE
case report guidelines

CARE Checklist of information to include when writing a case report

(cc) BY-NC-ND

Topic	Item	Checklist item description	Reported on Line
**Title**	1	The diagnosis or intervention of primary focus followed by the words "case report"	Page 1, Line 1
**Key Words**	2	2 to 5 key words that identify diagnoses or interventions in this case report, including "case report"	Page 4, Line 8
**Abstract (no references)**	3a	Introduction: What is unique about this case and what does it add to the scientific literature?	Page 3, Line2-5
	3b	Main symptoms and/or important clinical findings	Page 3 Line 6-22
	3c	The main diagnoses, therapeutic interventions, and outcomes	Page 3, Line13-22
	3d	Conclusion—What is the main "take-away" lesson(s) from this case?	Page 4, Line 2-6
**Introduction**	4	One or two paragraphs summarizing why this case is unique (**may include references**)	Page 5
**Patient Information**	5a	De-identified patient specific information.	Page 6 Line 2-8
	5b	Primary concerns and symptoms of the patient.	Page 6, Line 8-14
	5c	Medical, family, and psycho-social history including relevant genetic information	Page 6, Line 2
	5d	Relevant past interventions with outcomes	Page 6, Line 5-7
**Clinical Findings**	6	Describe significant physical examination (PE) and important clinical findings.	Page 7, Line 1-7
**Timeline**	7	Historical and current information from this episode of care organized as a timeline	Page 7 Line 1
**Diagnostic Assessment**	8a	Diagnostic testing (such as PE, laboratory testing, imaging, surveys).	Page 7, Line 8-11
	8b	Diagnostic challenges (such as access to testing, financial, or cultural)	Page 7, Line13-22
	8c	Diagnosis (including other diagnoses considered)	Page 8 Line 8-10
	8d	Prognosis (such as staging in oncology) where applicable	N/A
**Therapeutic Intervention**	9a	Types of therapeutic intervention (such as pharmacologic, surgical, preventive, self-care)	Page 8, Line 12-15
	9b	Administration of therapeutic intervention (such as dosage, strength, duration)	Page 8, Line 15
	9c	Changes in therapeutic intervention (with rationale)	N/A
**Follow-up and Outcomes**	10a	Clinician and patient-assessed outcomes (if available)	Page 8, Line 22
	10b	Important follow-up diagnostic and other test results	Page 9, Line 2-5
	10c	Intervention adherence and tolerability (How was this assessed?)	Page 9, Line 6-8
	10d	Adverse and unanticipated events	Page 9, Line 6-8
**Discussion**	11a	A scientific discussion of the strengths AND limitations associated with this case report	Page 9, Line11-20
	11b	Discussion of the relevant medical literature **with references**.	Page 10-13
	11c	The scientific rationale for any conclusions (including assessment of possible causes)	Page13,Line15-21
	11d	The primary "take-away" lessons of this case report (without references) in a one paragraph conclusion	Page13, Line15-21
**Patient Perspective**	12	The patient should share their perspective in one to two paragraphs on the treatment(s) they received	N/A
**Informed Consent**	13	Did the patient give informed consent? Please provide if requested	Yes ☑ No ☐
